# Effectiveness of Interventions to Promote Medication Adherence in Schizophrenic Populations in Thailand: A Systematic Review

**DOI:** 10.3390/ijerph19052887

**Published:** 2022-03-02

**Authors:** Suchanart Inwanna, Cherdsak Duangchan, Alicia K. Matthews

**Affiliations:** 1 Department of Population Health Sciences, College of Nursing, University of Illinois Chicago, Chicago, IL 60612, USA; cduang2@uic.edu (C.D.); aliciak@uic.edu (A.K.M.); 2 Ramathibodi School of Nursing, Faculty of Medicine Ramathibodi Hospital, Mahidol University, Bangkok 10400, Thailand; 3 Faculty of Nursing, HRH Princess Chulabhorn College of Medical Science, Chulabhorn Royal Academy, Bangkok 10210, Thailand

**Keywords:** medication adherence, interventions, cognitive-behavioral therapies, motivational interviewing, counseling, schizophrenia, Thailand

## Abstract

Medication non-adherence is a leading cause of poor treatment outcomes among Thai patients with psychiatric disorders and creates challenges for psychiatric nurses. This systematic review synthesized research on intervention effectiveness for antipsychotic medication adherence in Thai schizophrenic populations. Following PRISMA guidelines, searches were completed in seven databases, including PubMed, PsycINFO, CINAHL, Web of Science, Scopus, ThaiJO, and Google Scholar. No restriction dates were used. Screening and extraction of data were performed systematically. Eligible studies consisted of nine quasi-experimental and two randomized control trial studies. The Joanna Briggs Institute Meta-Analysis of Statistics Assessment and Review Instrument (JBI-MAStARI) was used to assess the studies’ methodological quality. This review revealed that individual counseling combined with cognitive-behavioral therapy (CBT) and motivational interviewing (MI) techniques improved and maintained medication adherence behavior over time. Post-testing after intervention completion and at 3- and 6-month follow-ups showed that treatment group participants were more adherent than control group participants (*p* < 0.01). These findings suggest that incorporating CBT and MI into clinical practice can enhance medication adherence behavior. Booster session efficacy for reinforcing and sustaining adherence should be investigated. Greater rigor is warranted in future intervention studies based on a quality appraisal of previous studies.

## 1. Introduction

According to the World Health Organization (WHO), psychiatric disorders represent a leading cause of morbidity and mortality worldwide [1]. Globally, approximately 970 million individuals suffer from a diagnosed psychiatric disorder [2]. In Thailand, data from the National Epidemiology of Psychiatric Comorbidity Survey indicated that 14.3% of Thai nationals had been diagnosed with a psychiatric disorder [3]. The most commonly diagnosed psychiatric disorders in Thailand include anxiety disorder, major depressive disorder, substance-use disorders, and schizophrenia, respectively [4]. In 2010, the prevalence of schizophrenia in the Thai population aged 15 to 59 years was approximately 8.8 per 1000 people [5].

Psychiatric disorders are associated with several negative consequences, such as reduced individual well-being, increased family burden, and barriers to employment and financial stability [6,7]. Numerous psychopharmacological, psychosocial interventions, case management, problem-solving, and motivational interviewing treatments have been identified that improve patients’ symptoms and overall functioning associated with severe psychiatric disorders [8]. However, uptake of and adherence to efficacious psychopharmacological approaches remain low, with only about 50% of patients with severe psychiatric disorders such as schizophrenia and bipolar disorder reporting adherence to prescribed medications and other treatments [9,10]. In Thailand, psychiatric nurses are primarily responsible for providing patient education and promoting adherence to psychotropic medications during hospitalization and discharge planning [11]. However, the Thai nurses association currently does not have clinical practice guidelines that specify best practices for improving medication adherence. As such, a review of the extant research and best practices associated with medication adherence approaches is needed to inform clinical practice guidelines for psychiatric nurses in Thailand [12].

The World Health Organization has defined medication adherence as “the extent to which a person’s behavior—taking medications, following diets, and/or executing lifestyle changes, corresponds with agreed recommendations from a health care provider” [13]. Moreover, the European Society for Patient Adherence, COMpliance, and Persistent (ESPACOMP) Medication Adherence Reporting Guideline (EMERGE) defined medication adherence as “the process by which patients take their medications as prescribed” and conceptualized medication adherence as consisting of three distinct phases, including initiation, implementation, and persistence [14]. Furthermore, EMERGE asserted that medication non-adherence could arise during any of the three phases. This conceptualization can assist researchers in operationalizing, measuring, and analyzing medication non-adherence [14].

The prevalence of medication non-adherence is high in psychiatric populations, and non-adherence is the leading cause of poor treatment outcomes [8,9,10,12]. In Thailand, medication non-adherence is highest among patients with bipolar disorder (64.3%) [12], followed by schizophrenia (24%) and major depressive disorder (23%) [15,16]. Poor treatment outcomes include an increase in psychotic symptoms, disease relapse, and rehospitalization, as well as higher treatment costs [17,18,19]. The factors related to medication non-adherence in psychiatric patients can be categorized into several groups, including patient-, clinical-, treatment-, and environment-related factors. Patient-related factors refer to socioeconomic factors and other individual-level characteristics influencing medication adherence [12,20]. Clinical-related factors are clinical characteristics, such as illness severity, age at onset, and duration of illness [19,20,21]. Treatment-related factors include prescribed medicines, the number of medications taken per day, and medication side effects [21,22,23]. Finally, concerning medication adherence, environment-related factors are typically related to the adequacy of social support [21,22,23]. Patient-level barriers related to treatment adherence include stigma, negative treatment attitudes, adverse side-effects, low health literacy, financial issues, and lack of social support [12,24].

In Thailand, clinical management of psychiatric disorders includes prescribing psychotropic drugs combined with psychosocial interventions, such as psychoeducation [25,26], cognitive-behavioral therapy [27], and family-focused therapy [25,28,29,30,31]. Despite the availability of effective treatment approaches, medication non-adherence remains a significant obstacle to managing psychiatric disorders. For this reason, we initially aimed to review and synthesize the existing literature related to increasing medication adherence in psychiatric populations in Thailand. However, most existing studies were found to focus on schizophrenia, with only a few addressing other psychiatric disorders, such as major depressive disorder and autism spectrum disorder; moreover, these latter studies failed to meet our review’s inclusion criteria. Therefore, our systematic review focused on examining the effectiveness of interventions for promoting antipsychotic medication adherence, specifically in schizophrenic populations.

## 2. Materials and Methods

### 2.1. Design

**Figure 1:**
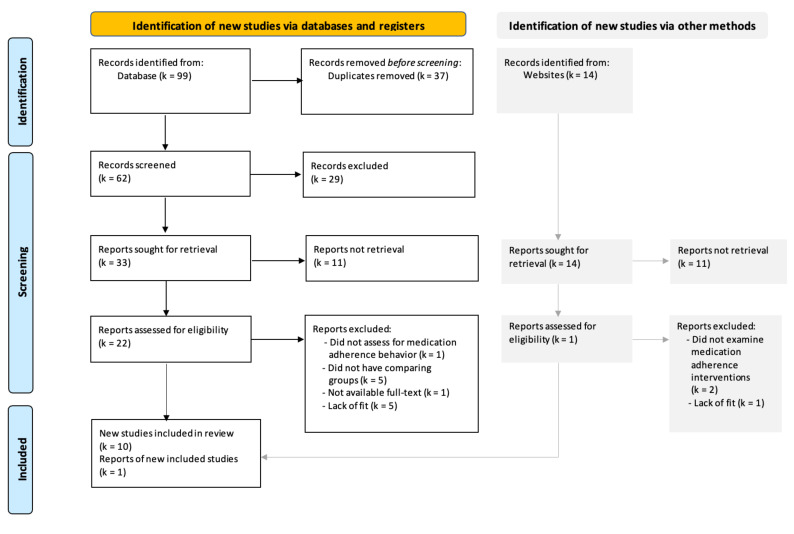
PRISMA 2020 flowchart of the study selection process of literature search.

This systematic review was conducted following the Preferred Reporting Items for Systematic Reviews and Meta-Analysis (PRISMA) Guidelines [32]. These guidelines consist of a 27-item checklist and the revised flowchart (Figure 1). However, this systematic review is not registered in PROSPERO.

### 2.2. Search Methods for Identification of Studies

Medical Subject Headings (MeSH) and keywords were constructed based on the purpose of the review. A search for relevant studies was completed in six databases: PubMed, PsycINFO, CINAHL, Web of Science, Scopus, and ThaiJO. In addition, a manual search was conducted in Google Scholar to identify additional suitable studies [33]. The search terms employed were *medication adherence/non-adherence/non-adherence*, *medication compliance/non-compliance/non-compliance*, *treatment adherence/non-adherence/non-adherence*, *mental disorders*, *mental illness*, *psychiatric disorders*, and *Thailand or Thai population*. The authors implemented search strategies with the guidance of an experienced health science librarian. No restriction dates were used to identify all available studies conducted in Thailand.

### 2.3. Eligibility Criteria

The studies to be included in this systematic review had to meet the following inclusion criteria: (1) focused on interventions related to medication adherence for patients with psychiatric disorders and/or their families; (2) reported on participants diagnosed with severe psychiatric disorders, including schizophrenia, bipolar disorder, or major depressive disorder, and received psychotropic drug treatment; (3) compared two or more groups using an experimental research design; (4) included medication adherence measurements as either primary or secondary outcomes; (5) conducted the study in Thailand; (6) the article was published in English, or the Thai language; (7) full-text was available; and (8) the interventions were developed and implemented by nurses. Unpublished studies, theses, dissertations, book chapters, and conference articles were excluded from the review.

### 2.4. Data Analysis

The search generated 99 articles from the search databases and 14 articles from Google Scholar, of which 37 were eliminated due to duplications. After screening the titles and abstracts of 76 articles, 23 studies remained. Two authors (S.I. and C.D.) who are bilingual in Thai and English independently reviewed the full text of these studies based on the inclusion and exclusion criteria. Subsequently, 11 eligible articles were included in this review. Data extracted from these studies were summarized based on the matrix method [34] using a narrative synthesis approach [35]. Table 1 summarizes the characteristics of the 11 studies, including their author(s), year of publication, purpose, study design, sample, setting, intervention approach, measures, findings, and quality appraisal score.

### 2.5. Quality Appraisal

The authors evaluated all 11 eligible studies for methodological quality using the Joanna Briggs Institute Meta-Analysis Assessment and Review Instrument (JBI-MAStARI) [46], which provides 9- and 13-item critical appraisal checklists for quasi-experimental and randomized control trial (RCT) studies, respectively. Using the 9-item checklist, the authors evaluated nine quasi-experimental studies for issues involving cause and effect, comparison of groups to detect selection bias, comparison of groups to confirm consistency of treatment, use of a control group, measurement outcomes, follow-up and dropout, measurement consistency, reliability of outcome measurement, and statistical analysis. The two RCT studies were appraised using the 13-item checklist, which addressed matters, such as participant randomization, group allocation concealment, group similarity, blinding, identical treatment, dropout, intention-to-treat analysis, outcomes measured, and statistical analysis. Differences of opinion between the two reviewers (S.I. and C.D.) were resolved through discussion with a third reviewer (A.K.M.) experienced in systematic reviews. Based on their total quality appraisal scores, the 11 studies were categorized for risk of bias into three groups: (1) low risk (met all criteria), (2) moderate risk (one or more criteria are not met, (3) high risk (one or more criteria are unmet) (Table 1) [47].

## 3. Results

Of 113 articles identified, 11 articles meeting the criteria were included in this systematic review. The publication years of the articles ranged from 2007 to 2020, with five articles published in the past 5 years. Nine studies employed quasi-experimental methods, and two were randomized controlled trials (RCT). The sample sizes of the 11 studies ranged from 24 to 100 participants and totaled 589 participants (mean sample size = 53.5). All participants had been diagnosed with schizophrenia.

### 3.1. Quality Appraisal

We applied the JBI-MAStARI tool to appraise the quality of the 11 studies and found that the risk of bias varied from moderate (one study) to high (10 studies). All nine quasi-experimental studies were appraised as having poor quality due to a high risk of bias. The most common factor reducing the studies’ quality was the failure to use multiple measurements of outcomes both pre-and post-intervention. Another factor related to the high risk of bias was unclear reporting about the similarity of participants included in the intervention and control groups [36,37,38,39,40,41]. As for the two RCT studies, one had a moderate risk of bias because of unclear reporting about blinding [43]. The particular concerns regarding this study were that it provided insufficient information about blinding of (1) participants as to which group they were assigned to (2) participants regarding intervention delivery to prevent them from knowing which group received the intervention or treatment as usual (the control group), and (3) researchers to prevent them from knowing participants’ group assignments to avoid biased assessment of outcomes. The other RCT study was appraised as weak in quality due to the high risk of bias [44]. In that study, the researchers provided no information about the blinding of participants regarding intervention delivery.

### 3.2. Sample Population

All studies included patients diagnosed with schizophrenia. Most studies (n = 8) recruited participants were males [25,38,39,41,42,43,44,45], aged either 18 and older or 20 and older [25,36,37,39,40,42,43,44,45]. Two studies did not mention their participants’ ages [38,41]. In eight studies, participants exhibited mild psychotic symptoms; the Brief Psychiatric Rating Scale (BPRS) was used to assess psychotic symptom severity as an inclusion criterion [25,37,38,39,40,41,42,45]. Additionally, one study measured stable psychotic symptoms using the Health of the Nation Outcome Scales (HoNOS); an HoNOS score ≤ of 2 was an inclusion criterion [42]. Finally, in five studies, a prior history of medication non-adherence was an inclusion criterion [25,36,37,39,41].

### 3.3. Treatment Settings and Initiation

All 11 studies were conducted in hospitals in Thailand. Six studies were conducted in psychiatric hospitals [37,40,42,44,45], and four were performed in non-psychiatric hospitals [25,38,39,41]. One study did not clearly identify the hospital setting [43]. Seven studies were performed in an intervention setting within hospital Inpatient Departments (IPD) (63.6%), three studies within Psychiatric Out-Patient Departments (OPD) (27.3%), and one study within the community (9.1%). In one of the studies set in an IPD, post-discharge sessions were held via telephone to assess participants’ health status, discuss any problems, and solve problems [45].

Nine of the 11 studies did not report the timing of treatment initiation. The remaining two studies identified the initial treatment timing as 1 week before hospital discharge [25,45].

### 3.4. Intervention Approaches

All the studies were nurse-led and offered either individual or group programs as face-to-face interventions. The number of program sessions ranged from four to 10, and the average number of sessions was six. The timing of the sessions ranged from 15 to 90 min, with an overall average of 50 to 60 min per session. Various psychosocial approaches were employed in the studies, including psychoeducation [25,45], counseling [41], adherence therapy [42,43,44], a family-centered approach [39], and motivational interviewing [40].

Three studies employed theory to guide programs to enhance medication adherence behavior [36,37,38]. Suntarowit et al. [36] employed Pender’s Health Promotion Model to modify patients’ behavior by encouraging awareness about taking care of themselves. Luangmongkhonchai & Lueboonthavatchai [38] applied Bandura’s Self-Efficacy Theory to achieve patient behavioral change by improving their self-efficacy in medication adherence. Tanabodee-tummajaree & Themrasi [37] used the necessity-concern framework to conduct the intervention, believing that medication adherence is influenced by treatment beliefs and concern about taking medicines. In addition, one study developed its program based on consideration of effective psychoeducation activities identified in a systematic review and adjusted the activities to make them suitable for Thai patients with schizophrenia [45]. In the remaining eight studies, interventions were developed based on a literature review and were not theory-driven [25,37,39,40,41,42,43,44].

### 3.5. Control Conditions

Study investigators included a comparison or control group in the 11 studies reviewed. The control group sizes in the studies ranged from 12 to 50 participants. In eight studies [36,37,38,39,40,43,44,45], the control conditions were described using somewhat different terms, such as routine nursing care, regular nursing care, treatment as usual, or standard care. The nursing care provided depended on the daily activity schedule in each setting. As part of the control conditions, two studies provided physical and mental well-being assessments, counseling, and advising [39,40]. Six studies provided group psychoeducation to enhance knowledge about schizophrenia, psychotropic drugs, drug side effects, and management of side effects [36,37,38,40,43,45]. In addition, two studies included other psychosocial interventions—for instance, occupational, group, and recreation therapies [43,44]. Three studies did not describe any aspect of the control conditions [25,41,42].

### 3.6. Treatment Completion and Retention

After intervention implementation in the 11 studies, all participants in the experimental groups completed the interventions, resulting in 100% treatment completion. Several studies measured outcomes at different follow-up periods, and participant retention for follow-up assessment varied from 87.5% to 100%. As examples, the retention rate immediately and less than 1 month after intervention completion ranged from 87.5% to 100% [25,36,37,38,39,40,41,43,45], at 3 months post-intervention was 100% [37,41], and at 6 months post-intervention ranged from 97.7% to 100% [37,41,44]. Only two studies reported participant loss during post-intervention follow-up [40,43]. One of those studies reported that lost participants had died or moved abroad [43], and the other study did not explain participant attrition [44].

### 3.7. Primary and Secondary Outcomes

All studies measured outcomes based on self-reporting. Eight studies measured medication adherence behavior [25,36,37,38,39,40,41,42] as their primary outcome, while two studies measured psychotic symptoms [43,44]. The remaining study measured attitude toward drugs as the primary outcome [45]. The intervention groups had significantly higher mean scores for medication adherence (*p* < 0.05) [25,36,37,38,39,40,41] and drug attitude (*p* < 0.05) [41,45] and lower mean scores for psychotic symptoms (*p* < 0.05) [41,43,44] than the control groups. In general, the interventions improved medication adherence behavior, instilled more positive attitudes toward medications, and decreased psychotic symptoms.

Among the studies having secondary outcomes, intentions to follow-up and actual follow-up after hospital discharge were measured in two studies [38,45]. After the intervention completion, the intervention group had significantly higher intentions to follow-up than the control group (*p* < 0.05) [38]. Moreover, the intervention group had a higher proportion of attendance for the first post-discharge follow-up appointment than the control group (*p* < 0.05) [45]. In addition, three studies measured attitudes toward drugs as their secondary outcomes. Two of these studies reported that the intervention group had a more positive attitude toward medication adherence than the control group [42,43]. In contrast, von Borman et al. reported no significant difference in attitude toward drugs between the two groups (*p* > 0.05) [44].

### 3.8. Measurement of Medication or Treatment Adherence

Several outcome measures evaluated antipsychotic medication adherence behavior, including standardized tools and instruments, developed based on theory. Ten studies used standardized tools to measure medication adherence either as their primary or secondary outcomes, including [25,37,38,39,40,41,42,43,44,45], Medication Adherence Scale, Brief Psychotic Rating Scale, Drug Attitude Inventory, and Brief Illness Perception Questionnaire. The majority of standardized measures used in eligible studies had been measured in prior research with high reliability. Moreover, Chiyajan and her team systematically translated the Drug Attitude Inventory-10 in Thai using back-translation procedures. They tested for psychometric properties among the Thai population before using it in their study [45]. Several outcome measures were initially developed in Thai and were widely used in nursing research, such as the Medication Adherence Behavior Scale [25,38] and Compliance Behaviors Assessment Scale [41,42]. One study developed an outcome instrument, the Health Behavior for Medication Adherence Assessment Form, based on Pender’s Health Promotion Model; this instrument’s psychometric properties were tested (Cronbach’s alpha = 0.81) [36]. As to the validity of medication adherence measures, only five studies reported the content validity of the measures used, which was determined by expert judgment [25,36,39,41,45]. Two of those studies reported the content validity index (CVI) of the measures used as 0.89 [25,39], and one of the studies reported the item objective congruence (IOC) index of the Health Behavior for Medication Adherence Assessment Form as 1 [36].

### 3.9. Effectiveness of Medication Adherence Interventions

Eight studies assessed antipsychotic medication adherence outcomes immediately post-intervention or less than one month after intervention completion. In addition, two studies measured these outcomes at three time-points after the intervention, including immediately post-intervention, in the short term (≤3 months), and the intermediate-term (4–6 months) [37,41]. Only one study used a single intermediate-term outcome assessment 26 weeks after intervention completion [44]. No studies reported long-term medication adherence outcomes (≥12 months).

**Figure 2:**
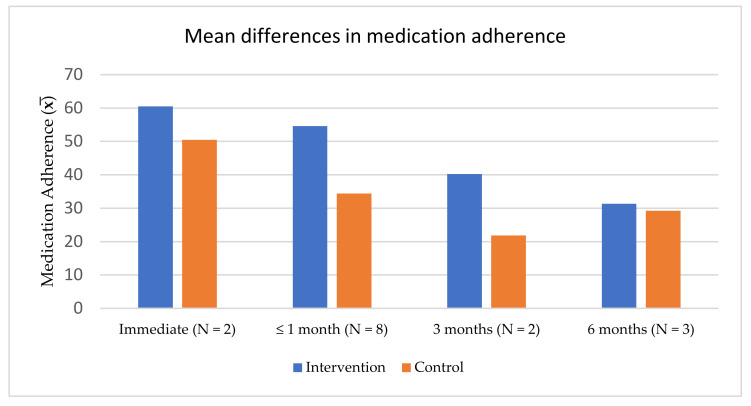
Differences in mean medication adherence scores between intervention and control groups.

Figure 2 summarizes medication adherence outcomes across the 11 studies reviewed. Overall, the mean scores for medication adherence were higher in the intervention groups compared to the control groups at all time-points. However, the medication adherence scores for the intervention groups tended to decrease over time; for example, the mean medication adherence score was 60.47 at the immediate time-point [36,41] and decreased dramatically (to a mean of 31.31) by the 6-month time-point [37,41,44].

**Figure 3:**
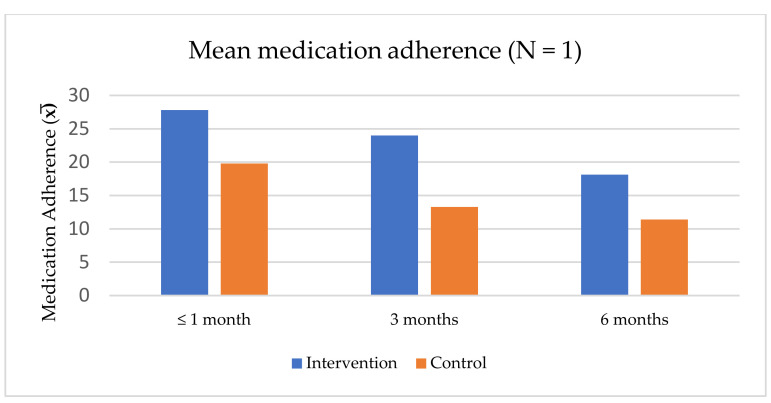
Mean medication adherence scores for a program promoting illness awareness and belief in medication [37].

Among the 11 studies, only two examined intervention effectiveness over time (at 1, 3, and 6 months post-intervention). Of those two studies, Tanabodee-tummajaree and Themrasi found that a program promoting illness awareness and belief in medication resulted in a consistently higher medication adherence score in the intervention group than in the control group [37]. However, the medication adherence scores for both groups decreased over time (Figure 3).

**Figure 4:**
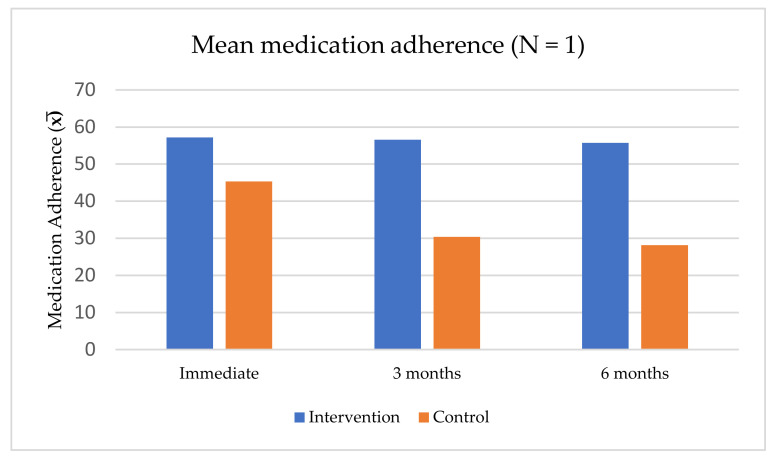
Mean medication adherence scores for individual counseling programs based on cognitive-behavioral therapy and motivational interviewing techniques [41].

In contrast, Wachiradilok and Rungreangkulkit found that an individual counseling program based on CBT and MI techniques produced overall medication adherence scores better than the control group at all three time-points and the intervention’s effectiveness was maintained over time [41] (Figure 4).

## 4. Discussion

The purpose of this systematic review was to evaluate the extant literature on medication adherence among patients with psychiatric disorders. While there is a clear need to examine the effectiveness of interventions intended to promote medication adherence among the various populations of psychiatric patients, most published studies of such interventions conducted in Thailand were limited to patients with schizophrenia. The few studies of interventions for patients with major depressive disorder or autism spectrum disorder either did not measure medication adherence as an outcome or did not involve a nurse-led intervention, thus failing to meet our review’s inclusion criteria. Consequently, we focused our systematic review on synthesizing and analyzing available evidence regarding the effectiveness of antipsychotic medication adherence interventions for Thai patients with schizophrenia. A total of 11 studies employing quasi-experimental and randomized control trial research designs met the eligibility criteria for the review. The overall quality of the intervention studies was mixed, ranging from moderate (9.09%) to high risk of bias (90.91%). Among the quasi-experimental studies, the main factor reducing methodological rigor was the lack of multiple pre-and post-intervention measurements. Although all these studies measured pre-and post-intervention outcomes, they employed only a single measurement pre-intervention; a few studies used multiple measurements post-intervention. The use of multiple measurements before an intervention makes it possible to explore explanations (cause) other than the intervention for the outcomes (effect) [46]. Additionally, implementing multiple measurements after an intervention allows investigation of changes in outcomes in each group (intervention and control) and comparison of these changes between groups [46]. For the RCT studies, the factors associated with reduced methodological rigor were the lack of blinding of participants and researchers.

Of the 11 studies reviewed, almost all reported that the interventions enhanced medication adherence behavior compared to control groups; two of these studies measured intervention effectiveness over time, and one found that benefits were maintained over time. In only one study, the intervention showed no improvement in medication adherence [44].

In all the reviewed studies, patients with schizophrenia were the target population for psychiatric nurse-led interventions in hospital-based psychiatric units, including inpatient and outpatient departments and a community setting. Most interventions targeted hospitalized patients in inpatient and outpatient departments; only one study included additional “booster” follow-up sessions via telephone after discharge from the hospital [45]. Notably, only two interventions were applied a week before hospital discharge [25,45]. However, interventions should be provided to patients receiving antipsychotic drugs for the first time to encourage medication adherence behaviors and promote conformance with healthcare professionals’ recommendations [37].

The primary focus of all the reviewed studies was medication adherence behavior in both intervention and control groups. The control condition included various routine nursing activities that depended on each department’s daily schedule in all the studies. These activities included assessing physical and mental illness, administering medication, providing treatment counseling, and implementing psychosocial interventions, such as psychoeducation, occupational therapy, group therapy, and recreation therapy. Several supportive interventions were available to address medication non-adherence and were generally effective. Further, one of the two studies examining intervention effectiveness over time found that effectiveness was maintained for at least six months [41]. In addition, some studies found that social support from family played an essential role in achieving an advantageous outcome in that family members encouraged patients to take their medication regularly [25,39].

Our review revealed that individual counseling involving a combination of CBT and MI techniques improved medication adherence behavior compared to the control group and maintained medication adherence over time. In general, this intervention had five steps: building a nurse–patient relationship, identifying patients’ treatment beliefs and hesitation to take medication, restructuring cognitive abilities, providing patients with practice in managing their medication, and allowing family members to practice expressing their emotions to patients [41]. The family involvement included in the intervention proved beneficial in improving patients’ medication adherence behavior [41]. Application of cognitive-behavioral strategies in an intervention can address distorted beliefs and negative perceptions about taking the psychotropic medicines needed for patient treatment [48]. Moreover, MI techniques are employed to explore and resolve patients’ ambivalence toward psychiatric drug-taking, help patients perceive the importance of medication adherence, and instill greater motivation to adhere to the medication regimen. In addition, counseling employing a combination of cognitive-behavioral and MI strategies is often used to change and improve medication adherence behavior patterns and to maintain long-term medication adherence [8,41]. Previous studies have provided additional evidence that the combination of cognitive-behavioral and MI methods is evidence-based and highly effective for improving medication adherence behavior and reducing relapse and rehospitalization rates among patients with schizophrenia [49].

Additionally, we found that the overall effectiveness of counseling combining cognitive-behavioral and MI strategies was consistent with the level of medication adherence. That effectiveness was maintained over time but still decreased slightly [41]. This decrease may be due to various confounding factors that might affect patients’ behaviors after receiving the interventions. Such factors could include patients’ lack of confidence in managing drug side-effects, stress, lack of problem-solving skills, and perceived stigma regarding their condition. To achieve better medication adherence outcomes, nurse interventionists in hospitals and community-based settings should provide follow-up booster sessions to help patients with schizophrenia contend with confounding factors and reinforce medication adherence behavior. By doing so, nurses can enhance the long-term effectiveness of their interventions.

### 4.1. Implications for Future Research and Nursing Practice

Overall, the studies reviewed showed a moderate to high risk of bias, preventing solid conclusions based on the study results. Researchers studying medication adherence interventions should make every effort to maximize methodological quality to reduce the risk of bias. For example, an experimental research design with an appropriate design framework, multiple pre-and post-intervention measurements of outcome variables, and inclusion of similar participants in the intervention and control groups would increase the credibility of the research and reduce the risk of methodological bias. In addition, ensuring that an RCT includes blinding participants and researchers as to intervention delivery would improve the methodological quality of such research.

Furthermore, the studies used several measures to assess medication adherence behavior. Previous research has reported no gold standard for subjective measurement of medication adherence behavior via self-report rating scales. Therefore, future studies that subjectively evaluate medication adherence behavior and adherence attitudes should employ multiple well-established tools to account for individual measures’ limitations. Examples of widely used self-report measures include the Brief Adherence Rating Scale, Morisky Adherence Rating Scale, Drug Attitude Inventory, and Medication Adherence Rating Scale [50]. Additionally, objective methods should be considered for measuring medication adherence behavior; for instance, the gold standard for monitoring pill-taking is to apply technology-supported methods such as Medication Event Monitoring or smart pill-boxes [50]. Because perfect tools to measure medication adherence do not exist, standardized self-report scales and objective technology-based methods should ideally be combined in clinical studies to ensure accurate assessment of medication adherence behavior [51].

Finally, our review revealed that a combination of cognitive-behavioral and motivational interviewing interventions could maintain antipsychotic medication adherence behavior over time. For this reason, we recommend incorporating these approaches into routine psychiatric nursing practice in both hospital and community-based settings. In addition, we found that delivering early behavioral intervention was associated with better adherence to medication regimens over time. Consequently, we recommend that medication adherence behavioral intervention be provided to patients as early as possible in the treatment process. Furthermore, given the decline in medication adherence behavior frequently observed over time, psychiatric nurses should provide booster sessions to help sustain long-term medication adherence. Additionally, psychiatric nurses are well-positioned to play a leading role in medication adherence interventions, but they will need specialized training in applying cognitive-behavioral and motivational interviewing techniques before implementing such strategies in their clinical practice.

### 4.2. Limitations

Several limitations should be considered when interpreting the results of this systematic review. After applying an established appraisal tool to examine the quality of the studies, we found that a moderate to high risk of bias compromised the studies’ methodological quality. Specifically, 10 studies were assessed as having high risk and one with moderate risk of bias. For the quasi-experimental studies, the principal factors contributing to such risk were the lack of multiple pre-and post-intervention measurements of outcome variables and lack of homogeneity between the intervention and control groups. The two RCT studies did not adequately report on the blinding of participants and researchers with respect to intervention delivery. This is a problem because lack of blinding can lead to faulty estimation of intervention outcomes [52].

Another potential limitation of this review involves the included studies’ failure to specify the subtype of schizophrenia diagnosed in their patient samples. The International Classification of Diseases (ICD-10) identified various subtypes of schizophrenia, including paranoid, hebephrenic, catatonic, undifferentiated, post-schizophrenic depression, residual, simple, other schizophrenia, and schizophrenia unspecified [53]. However, the ICD-11 and the Diagnostic and Statistical Manual of Mental Disorders (DSM-V) eliminated schizophrenia subtypes [54,55]. Furthermore, one study examined only male participants, limiting participant characteristics’ diversity [45]. Additionally, all the studies used self-report scales to measure medication adherence behavior. Previous research has suggested that self-report measures are more likely to produce inaccurate scores than other assessment methods, and self-report scales typically show high specificity but low sensitivity [56]. Moreover, the different types and components of interventions, program durations, numbers of sessions, outcome assessment periods, and outcome measures limited our ability to analyze and synthesize study results quantitatively. Finally, most of the studies measured outcomes only in the immediate and short term, and thus, we could not compare the effectiveness of interventions over time.

Despite these limitations, this review is the first to evaluate and compare interventions for improving antipsychotic medication adherence behavior among patients with schizophrenia in Thailand. As a result, our findings will provide helpful guidance for Thai psychiatric nurses who wish to apply a combination of cognitive-behavioral and motivational interviewing interventions to promote medication adherence behavior.

## 5. Conclusions

This systematic review indicates that adjunctive treatment employing cognitive-behavioral and motivational interviewing methods in counseling sessions has relatively high efficacy for enhancing medication adherence behavior among patients with schizophrenia. In addition, this combined intervention can maintain antipsychotic medication adherence behavior over time. These findings suggest the benefits of adopting this approach for routine psychiatric nursing care in hospital and community-based settings to promote medication adherence as a desirable behavior. Future studies should investigate the efficacy of supplementing intervention programs with ongoing booster sessions to maintain medication adherence behavior and evaluate interventions’ effectiveness in promoting long-term adherence. In such studies, researchers should ensure that their protocols minimize the risk of bias and maximize methodological quality.

## Figures and Tables

**Table 1:** Summary of Antipsychotic Medication Adherence Interventions for Thai Patients with Schizophrenia.

Authors Year	Aim	Design	Sample	Setting	Theory-Based	Intervention for Experimental Group	Regular Nursing Care for Control Group	Measurement	Findings	Quality Score
1. Koomala et al., 2020 [25]	To Investigate the effect of medication adherence promotion program in patients with Schizophrenia.	Quasi-experiment	24 patients with schizophrenia	Inpatient	1. Psychoeducation 2. Family involvement	No. of sessions: 5 (1 session/day) 1. Therapeutic relationship 2. Individual psychoeducation 3. Individual psychoeducation 4. Family psychoeducation 5. Skill practice Duration: 60–90 min/session Initiation: 1 week before discharge	N/A	1. Medication Adherence Behaviors Scale	- The program was effective in improving medication adherence. - At 2-week follow-up, the experimental group had a significantly different mean score on medication adherence (*p* < 0.001). - The experimental group had a significantly different mean score of medication adherence from the control group (*p* < 0.001).	High risk
2. Suntarowit et al. 2020 [36]	Compare medication adherence behavior before and after receiving a health promotion program and compare the program with a control group.	Quasi-experiment	68 patients with schizophrenia	Inpatient	1. Pender’s health promotion theory	No. of sessions: 6 (in 1 week) 1. Identifying patient’s characteristics 2. Identifying prior behavior 3. Identifying medication adherence & non-adherence 4. Problem-solving 5. Intention to medication adherence 6. Follow-up Duration: 60 min/session Initiation: N/A	1. Routine daily activity 2. Psychoeducation 3. Group therapy	1. Health Behavior for Medication Adherence Assessment Form	The program was effective in increasing health behavior for medication adherence. Immediately after the program completion and 1-month follow-up, the experimental group had significantly higher mean medication adherence scores than before (*p* < 0.001). - The experimental group had a significantly higher mean medication adherence score than the control group (*p* < 0.001).	High risk
3. Tanabo-deetumma-jaree & Themrasi, 2018 [37]	To develop and examine the program’s effectiveness to promote illness awareness and belief in taking medication on medication adherence.	Quasi-experiment	60 patients with schizophrenia	Inpatient	1. Necessary-concern framework	No. of sessions: 4 (in 2 weeks) 1. Illness awareness promotion 2. Belief in antipsychotic drugs 3. Common concerns and beliefs with antipsychotic drugs 4. Strategies to deal with barriers and individuals that affect medication adherence behavior Duration: 60 min/session Initiation: N/A	1. Psychoeducation (disease, medication, and self-care)	1. Brief Illness Perception Questionnaire 2. Beliefs in Taking Medication Questionnaire 3. Self-Medication Intake Record Form	The program was effective in increasing medication adherence behavior. - After program completion, the experiment group had a higher mean score of illness perception than the control group (*p* < 0.05). - After program completion, the experiment group had a lower belief score in taking medication than the control group (*p* < 0.05). - At 1,3, 6 months follow-up, the experiment group had a higher average day of medication adherence than the control group (*p* < 0.05).	High risk
4. Luangmongkhonchai & Lueboon thavatchai, 2011 [38]	To examine the effects of perceived self-efficacy promotion on medication adherence and follow-up an intention.	Quasi-experiment	40 patients with schizophrenia	Out-patient	1. Bandura’s self-efficacy theory	No. of sessions: 5 (in 4 weeks) 1. Emotional & physiological state stimulation 2. Role model 3. Foremost mastery experience 4. Verbal persuasion 5. Previous success experiences and being confident to take medicines and follow-up Duration: 60 min/session Initiation: N/A	1. Group psychoeducation	1. Medication Adherence Scale 2. Follow-up Intention Scale	The program effectively increased medication adherence behavior and intention to follow up. - After program completion (week 5), the experimental group had significantly higher mean scores of medication adherence and follow-up intention than that before (*p* < 0.05). - Experiment group had significantly higher mean scores of medication adherence and follow-up intention than the control group (*p* < 0.05).	High risk
5. Phuengnam & Uthis, 2017 [39]	To examine the effect of the family-centered empowerment program on medication adherence.	Quasi-experiment	40 patients with schizophrenia	Out-patient	1. Family-centered empowerment model 2. Empowerment 3. Families and family therapy	No. of sessions: 6 (in 4 weeks) 1. Building family’s awareness to medication adherence behavior 2. Developing family’s knowledge and skills 3. Recalling previous experiences on problem-solving 4. Practicing problem-solving skills 5. Exchanging experiences 6. Evaluation Duration: 90 min/session Initiation: N/A	1. Physical and mental assessment 2. Counseling 3. Advising	1. Drug Adherence Questionnaire	The program was effective in increasing medication adherence behavior. After program completion, the experimental group had significantly higher mean medication adherence scores than before (*p* < 0.05). - Experiment group had significantly higher mean scores of medication adherence than the control group (*p* < 0.05)	High risk
6. Uthaiphan & Dangdomyouth, 2013 [40]	To examine the effect of group motivational interviewing on medication adherence.	Quasi-experiment	40 patients with schizophrenia	Out-patient	1. Motivation interviewing 2. Stage of change	No. of sessions: 5 (in 4 weeks) 1. Behavior change motivation 2. Strengthen commitment to a target behavior change 3. Strengthen commitment to a target behavior change 4. Strengthen commitment to a target behavior change 5. Follow-up Duration: 60–90 min/session Initiation: N/A	1 Physical and mental assessment 2. Counseling 3. Advising 4. Group psychoeducation	1. Compliance Behaviors Assessment Scale	The program was effective in increasing medication adherence behavior. After program completion, the experimental group had significantly higher mean medication adherence scores than before (*p* < 0.05). - Experiment group had significantly higher mean scores of medication adherence than the control group (*p* < 0.05).	High risk
7. Wachiradilok & Rungreangkulkit, 2008 [41]	To examine the effect of individual counseling programs on treatment compliance.	Quasi-experiment	100 patients with schizophrenia	Out-patient	1. Motivational interviewing 2. Cognitive-behavioral technique	No. of sessions: 5 (1 session/week) 1. Therapeutic relationship 2. Seeking for beliefs and hesitation of treatment 3. Cognitive restructuring 4. Practicing medication administration skills 5. Family’s appropriate emotional expression Duration: 60 min/session Initiation: N/A	N/A	1. Compliance Behaviors Assessment Scale 2. Knowledge of Self Management Scale 3. Brief Psychotic Rating Scale	The program was effective in increasing treatment adherence behavior and self-management. - At program completion, 3-month, and 6- month follow-up, the experiment group had significantly higher mean scores of compliance behaviors and self-management knowledge than the control group (*p* < 0.01).	High risk
8. Eungsanran et al., 2012 [42]	To examine the effect of adherence group therapy program on treatment adherence, drug attitude, psychotic symptoms, and hospital readmission.	Quasi-experiment	60 patients with schizophrenia	Inpatient	1. Compliance therapy model 2. Motivational interviewing 3. Cognitive approaches	No. of sessions: 5 1. Engagement 2. Adherence Assessment 3. Eliminating barriers and hesitation on treatment adherence 4. Motivational interviewing and problem-solving 5. Improving self-confidence and practicing medication administration skills Duration: 60 min/session Initiation: N/A	N/A	1. Medication Adherence Scale 2. Brief Psychotic Rating Scale 3. Drug Attitude Inventory	The program was effective in increasing treatment adherence behavior and positive drug attitude. - At 1-month and 3- month follow-up, the experiment group had significantly higher mean scores of medication adherence (*p* < 0.05) and drug attitude (*p* < 0.05), but lower mean scores of psychotic symptoms (*p* < 0.001) than the control group. - No hospital readmission within three months.	High risk
9. Maneesakorn et al., 2007 [43]	To evaluate the effectiveness of adherence therapy on psychotic symptoms, general functioning, attitude towards and satisfaction with medication, and medication side effects.	Randomized Controlled Trial	32 patients with schizophrenia	Inpatient	1. Compliance therapy 2. Motivational interviewing 3. Cognitive approaches	No. of sessions: 8 (5 phases) (1 session/week) 1. Engagement 2. Assessment: problem, self-confidence, side effects experiences, beliefs, and attitudes 3. Rate readiness to take medicines 4. Intervention: problem-solving, timeline, exploring ambivalence, discussing beliefs and concerns, using medication in the future 5. Evaluation Duration: 15–60 min/session Initiation: N/A	1. Medication treatment 2. Occupational therapy 3. Group therapy 4. Recreation therapy	Primary: 1. Positive and Negative Syndrome Scale Secondary: 2. Global Assessment of Functioning Scale 3. Drug Attitude Inventory 4. Satisfaction with Antipsychotic Medication Scale 5. Liverpool University Neuroleptic Side Effect Rating Scale	The program effectively increased treatment adherence, attitude toward, and satisfaction with medication. - At nine-week follow-up, the experiment group had significantly more significant improvement on psychotic symptoms (*p* = 0.001), attitude towards (*p* = 0.001), and satisfaction with medication (*p* = 0.019) than the control group. - No significant difference was found in general functioning or side effects.	Moderate risk
10. von Borman et al., 2015 [44]	To examine the efficacy of adherence therapy on clinical outcomes.	Randomized Controlled Trial	70 patients with schizophrenia	Inpatient	1. Compliance therapy 2. Motivational interviewing 3. Cognitive approaches	No. of sessions: 8 sessions (6 exercises) (1 session/week) 1. Assessment: beliefs, problems, medication reconciliation 2. Problem-solving 3. Reviewing previous illness and treatment experiences 4. Seeking ambivalence to take medications 5. Examining beliefs about medication 6. Supporting life goals. Duration: 15–60 min/session Initiation: N/A	1. Medication, vocational, and recreation therapy 2. Outreach community psychiatric support	Primary: 1. Positive and Negative Syndrome Scale Secondary: 2. Global Assessment of Functioning Scale 3. Drug Attitude Inventory 4. Liverpool University Neuroleptic Side Effect Rating Scale	The program was effective in improving psychotic symptoms - At 26-week follow-up, the experiment group had significantly more significant improvement on psychotic symptoms than the control group (*p* < 0.05). - No statistically significant differences were found in attitude toward treatment, functioning, or side effects.	High risk
11. Chaiya-jan et al., 2009 [45]	To examine the effects of a psychoeducation program on attitude toward medication and compliance with a first follow-up appointment.	Quasi-experiment	55 patients with schizophrenia	Inpatient	1. Psychoeducation	No. of sessions: 10 sessions (1 session/day for the first six sessions, one session/week for the other four sessions) 1. Therapeutic relationship 2. Illness knowledge 3. Medication knowledge 4. Side effects management 5. Stress management 6. Symptom management 7–10. Assess health conditions, discuss the problem, and problem-solving Duration: -30 min/session (the first 6 sessions)-15–20 min/session (the other 4 sessions) Initiation: Last week of hospital stay	1. Psychoeducation 2. Reminding of regimens adherence and a follow up visit 3. Home visit	1. Drug Attitude Inventory	The program was effective in increasing positive attitudes toward medication. - After program completion, the experiment group had a significantly higher positive attitude toward medication (*p* < 0.05) and proportion of attendance for the first follow-up appointment than the control group (*p* < 0.05).	High risk

## Data Availability

No additional data. The article contains all the data.
